# Mechanism Research and Application for Ginsenosides in the Treatment of Hepatocellular Carcinoma

**DOI:** 10.1155/2023/7214037

**Published:** 2023-11-16

**Authors:** Nian Zhou, Feifei Mao, Shuqun Cheng

**Affiliations:** ^1^Tongji University Cancer Center, Shanghai Tenth People's Hospital, School of Medicine, Tongji University, Shanghai 200072, China; ^2^Eastern Hepatobiliary Surgery Hospital, Navy Medical University, Shanghai 200438, China

## Abstract

Ginsenosides, the main active pharmacological ingredients of ginseng, have been widely used for the treatment of numerous carcinomas. Hepatocellular carcinoma (HCC) is 3^rd^ leading malignant tumor in terms of mortality worldwide. Accumulating evidence indicates that ginsenosides play a vital role in the prevention and treatment of HCC. Ginsenosides can significantly improve the symptoms of HCC, and their anticancer activity is mainly involved in inhibiting proliferation and migration, inducing cell cycle arrest at the G0/G1 phase, promoting caspase-3 and 8-mediated apoptosis, regulating autophagy related to Atg5, Atg7, Atg12, LC3-II, and PI3K/Akt pathways, and lowering invasion and metastasis associated with decreased nuclear translocation of NF-*κ*B p65 and MMP-2/9, increasing IL-2 and IFN-*γ* levels to enhance immune function, as well as regulating the gut-liver axis. In addition, ginsenosides can be used as an adjuvant to conventional cancer therapies, enhancing sensitivity to chemotherapy drugs, and improving efficacy and/or reducing adverse reactions through synergistic effects. Therefore, the current manuscript discusses the mechanism and application of ginsenosides in HCC. It is hoped to provide theoretical basis for the treatment of HCC with ginsenosides.

## 1. Introduction

Hepatocellular carcinoma (HCC) is 3^rd^ highest cause of cancer mortality worldwide. It usually occurs in chronically damaged liver tissues [1, 2]. About 80-90% of HCC cases are related to cirrhosis or fibrosis. Meanwhile, infection with HBV and HCV, alcoholism, diabetes, nonalcoholic fatty liver disease (NAFLD), and hemochromatosis are well-recognized risk factors [3, 4]. Currently, there are many clinical treatments for HCC, including surgical resection, radiotherapy and chemotherapy, liver transplantation, radiofrequency ablation, transcatheter arterial chemoembolization (TACE), radioembolization, emerging systemic chemotherapy, and target-immune drugs. However, tumor recurrence and metastasis and chemotherapy resistance are common, so survival rate of HCC patients is very small [5, 6]. Therefore, we need to develop new drugs and technical means to further curb HCC occurrence and development.

**Figure 1:**
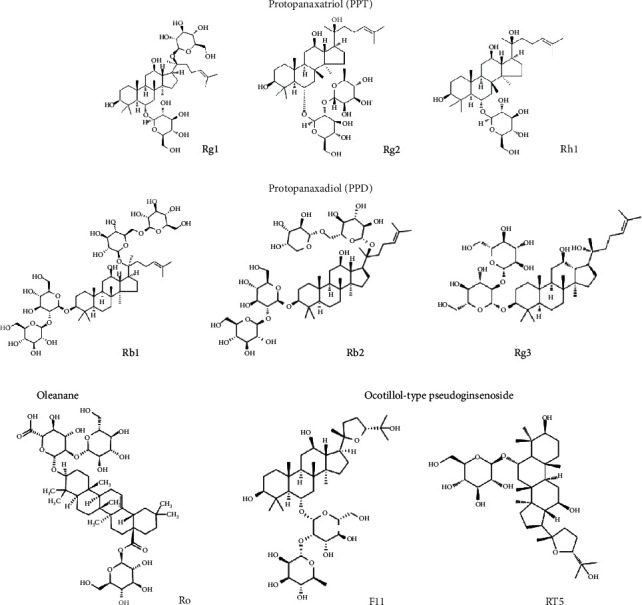
Classification of ginsenosides. There are four major different groups of ginsenosides including the protopanaxadiol (PPD), protopanaxatriol (PPT), oleanane, and ocotillol-type pseudoginsenoside.

Ginsenosides, commonly known as triterpenoid saponins in botany, are a class of steroid compounds. They are mainly derived from ginseng medicinal materials and are currently known to be present in 32 varieties of red ginseng and 24 varieties of white ginseng [7, 8]. According to their molecular structures, they are categorized into various types (Figure 1), such as protopanaxatriol (PPT), protopanaxadiol (PPD), ocotillol-type pseudoginsenoside, and oleanane. PPT types include Rg1, Rg2, Re, Rf, and Rh1, while PPD types include Rb1, Rb2, Rg3, Rh2, Rd, Rc, Rs1 and compound K (CK). The ocotillol-type pseudoginsenoside has F11, R1, R2, and RT5. The oleanane group includes oleanolic acid pentacyclic triterpenoid saponin (Ro) [9, 10]. Since ginsenosides are mostly complex substances, a series of measures have been taken to improve the absorption rate and bioavailability during the preparation process. By steaming, ginsenoside-Rh1 is frequently decomposed into 20(*S*)-Rg3, 20(*R*)-Rg3, Rk1, and Rg5. Meanwhile, 20(*S*)-Rh2, 20(*R*)-Rh2, Rk2, and Rh3 were transformed into 20(*S*)-PPD, 20(*R*)-PPD and Rg3 under *Bacteroides* and *Eubacteria* action, respectively [11–13]. Modern pharmacological studies have shown that ginsenosides have good antitumor, anti-inflammatory, antioxidant, antiapoptotic, and immunomodulatory properties [14, 15]. In addition, several investigations demonstrated antitumor properties and mechanisms of different types of ginsenosides through JNK/p53, Wnt/*β*-catenin, ERK, and other related pathways [16].

In recent years, numerous research demonstrated the therapeutic potential of ginsenosides for HCC. Hence, we concentrate on the mechanism research and combined application progress of ginsenosides as potential novel therapeutic options for HCC therapy.

## 2. Study on the Therapeutic Effect and Mechanism of Ginsenoside on HCC

Ginsenosides are a class of natural compounds that can be used as anticancer agents for HCC, and their anticancer effects have been extensively studied (Table 1). Therefore, the mechanisms of their anticancer effects are described in detail below.

### 2.1. Regulate the Cell Cycle, Proliferation, and Migration Ability of HCC

Normal cell cycle contains 4 phases: G0/G1 (gap), S (synthesis), G2 (gap), and M (mitosis), actively controlled by cyclin-dependent Kinase (CDK). Abnormal cell cycle progression in cancer is associated with uncontrolled cell proliferation; therefore, controlling cell cycle is one of the probable malignancy treatment techniques [17, 18]. 20(*S*)-Rh2 treatment of HepG2 and Hep3B cells resulted in suppression of cell proliferation, migration, and cell cycle arrest in G0/G1 phase stimulation. In addition, studies have shown that 20(*S*)-Rh2 could suppress ZESTe homolog 2 (EZH2) expression, which is an effective histone methyltransferase of histone 3 lysine 27 (H3K27me3) and has been identified as an oncogene in many malignant tumors. EZH2 overexpression is linked with worse prognosis of HCC [19]. Ginsenoside-Rg3 suppresses HCC proliferation and invasion by controlling the expression of long noncoding RNA HOX antisense intergenic (HOTAIR) involved in PI3K/Akt signaling pathway [20]. 20(*S*)-Rh2 interferes with the interaction of tumor-associated protein annexin A2 with signal transducers and activators of transcription 3(STAT3) and inhibits Tyr705 phosphorylation and subsequent transcriptional activity, resulting in negative regulation of four vascular endothelial growth factors (VEGFs), which substantially lowers improved growth and migration capacity of HUVECs coculture system [21]. Treatment of HepG2 and SK-Hep-1 cells with 20(*S*)-Rh2 inhibits cell proliferation and migration and induces cell cycle arrest in G0/G1 phase through the interaction between heat shock protein 90 alpha (HSP90A), a key regulatory protein related to HCC cancer, and its common partner cell division cycle control protein 37 (Cdc37) [22]. Meanwhile, the CDKN2A-2B gene cluster encodes three important tumor suppressor genes (p14, p15, and p16), and EZH2 enhances transcription of P14, P15, and P16 genes by lowering H3K27me3 modification in the promoter of CDKN2A-2B gene cluster loci. Therefore, 20(*S*)-Rh2 regulates CDKN2A-2B gene cluster transcription by targeting EZH2 and suppressing HCC proliferation and migration [19, 23].

### 2.2. Induced Apoptosis in HCC

Apoptosis, a form of programmed cell death, is a crucial way to regulate homeostasis by eliminating redundant and abnormal cells. Cancer is characterized by abnormal regulation of apoptosis. Consequently, triggering apoptosis is one of the most efficient cancer treatments [24]. Two major pathways regulate apoptosis: death receptor-mediated (extrinsic) and mitochondria-dependent (intrinsic) [25]. These pathways activate a variety of apoptotic proteases, which cleaves specific substrates and ultimately leads to cell death [26]. Caspases are a family of proteases that control apoptosis, including upstream promoter caspases, such as caspase-8 and 10, and downstream executive caspases, such as caspase-3 [27, 28]. In HepG2 cells, ginsenoside Rk1 treatment can significantly reduce telomerase reverse transcriptase (hTERT) and c-Myc mRNA expression levels, and Rk1 can also induce apoptosis by activating caspase-8 and 3. Moreover, Rk1 therapy increases extracellular regulated kinase (ERK) activity. These findings demonstrate, for the first time, Rk1 biological activity on HepG2 cell growth, suggesting that the antitumor activity mechanism of Rk1 is related to the synergistic effect among telomerase activity inhibition and apoptosis induction [29]. At the same time, ginsenoside-Rg3 attenuates tumor volume and the capability to generate vascularized network by stimulating apoptosis and suppressing angiogenesis, thus prolonging the survival of mice in orthotopic HCC model [30].

### 2.3. Regulation of Autophagy in HCC

Additionally, through cell cycle arrest and induction of apoptosis, autophagy can, in some cases, not only directly play a cancer suppressor role by eliminating damaged cells but also indirectly protect cells from carcinogenesis by maintaining genome stability and homeostasis through cytoprotection. Therefore, regulating autophagy could also be an approach for cancer therapy [31, 32]. Studies have shown that 20(*S*)-ginsenoside-Rg3 inhibits autophagy at a late stage by inhibiting maturation, fusion, or degradation stages, thereby achieving anticancer effects [33]. Autophagosome formation; increase of autophagy-related (ATG) 5, 12, beclin-1, and light chain-3 (LC3) B-II; and downregulation of p62 protein levels in a dose-dependent manner were observed in ginsenoside Rh2-treated HepG2 and Huh7 cells; ginsenoside Rh2 can also regulate autophagy and apoptosis by inhibiting PI3K/Akt pathway. Meanwhile, the detection outcomes expose that ginsenoside Rh2 inhibits HCC growth mainly through the coordination of autophagy and *β*-catenin signaling pathway [34].

### 2.4. Regulation of Invasion and Metastasis in the Development of HCC

Tumor invasion and metastasis is an important cause of treatment failure in cancer patients. Matrix metalloproteases (MMPs) are key enzymes involved in the destruction of extracellular matrix, basement membrane, and cancer cell invasion and metastasis [35, 36]. Studies have shown that Rh1 suppresses HepG2 migration and invasion in concentration- and time-dependent manner. Ginsenoside Rh1 can inhibit the activation of mitogen-activated protein kinases (MAPKs) ERK, JNK, and p38 in HepG2 cells, reduce transcriptional activity of MMP-1, and reduce expression and stability of activated protein AP-1 dimers c-fos and c-Jun, thereby producing antimetastasis activity [37]. Meanwhile, some studies not only confirm that ginsenoside CK could significantly inhibit the colony formation, adhesion, and invasion of HCC cells but also reveal that significant inhibition of spontaneous metastatic growth of HCC cells by CK is related to translocation of nuclear factor-*κ*B (NF-*κ*B) p65 and MMP-2/9 reduction [38].

### 2.5. Regulation of Immune Function

The immune system can eliminate malignant allogeneic cells through cellular immune regulation. However, these malignant nonself cells can evade immune surveillance and form tumors when immune function is low. Therefore, promoting cellular immunity through immune-stimulating drugs is a feasible way to inhibit tumor growth without harming the host [39]. Both 20(*S*)-Rg3 and 20(*R*)-Rg3 could significantly inhibit the growth of H22 xenografts and H22 tumor-bearing mice, with the inhibition rates of 23.6% and 40.9%, respectively. In addition, Rg3 increases the levels of Th1-type cytokines interleukin-2 (IL-2) and interferon-*γ* (IFN-*γ*) by stimulating the proliferation of lymphocytes induced by ConA and significantly enhances the cellular immune function of H22 hepatocellular carcinoma mice. Moreover, these results suggest that Rg3 can improve host cellular immunity and at least partially inhibit H22 tumor growth [40].

### 2.6. Inhibiting the Development of HCC by Targeting the Gut-Liver Axis

In HCC mouse model induced by dimethyl nitrosamine and CCl_4_, ginsenoside Rk3 can inhibit liver injury, fibrosis, and cirrhosis, as well as play a good antitumor role by reducing inflammatory cytokine expression, inducing tumor cell apoptosis, blocking cell cycle, and reducing the inflammatory response. It can also effectively increase *Bacteroidetes* abundance and reduce *Firmicutes* abundance in liver cancer mice. *Oscillibacter* is a bacterium that inhibits tumor growth and has anti-inflammatory characteristics [41]. Cancer-fighting bacteria *Akkermansia* protects against liver failure, lowers inflammation, and strengthens intestinal barrier. According to the results of subgenomic studies, HCC and liver cirrhosis patients' gut microbiomes have more pathogenic but fewer helpful bacteria [42, 43]. Some data show that supplementation of ginsenoside Rk3 significantly boosts beneficial bacteria abundance in HCC mice. *Ruminococcus* is closely related to nonalcoholic fatty liver disease and cirrhosis [44]. *Helicobacter* has also been implicated in HCC progression, and ginsenoside Rk3 therapy significantly decreases harmful bacteria abundance. Hence, the results show that ginsenoside Rk3 can successfully fix intestinal microbiota disorder in HCC mice and provide valuable insights for targeting gut-liver axis to inhibit HCC progression [45].

## 3. Combined Applications of Ginsenosides against Hepatocellular Carcinoma

In addition to being used alone, ginsenoside can also be used in combination with a variety of clinical drugs (Table 2), which cannot only reduce the toxic and side effects of chemotherapy drugs but also improve the therapeutic effect of its hepatocellular carcinoma. The mechanism of action is detailed below.

### 3.1. Ginsenoside Rg3 and Oxaliplatin

Oxaliplatin is a third-generation platinum-based antitumor drug with the advantages of high efficiency, few side effects, and broad anticancer spectrum [46]. Numerous studies have shown satisfactory results with oxaliplatin or in combination with other cytotoxic and/or targeted agents [47]. Proliferating cell nuclear antigen (PCNA) is clearly related to DNA synthesis and has a crucial function in cell proliferation regulation. Cyclin D1 contributes to liver cancer cell proliferation. In comparison with ginsenoside Rg3 group and oxaliplatin group, the study finds that combined application of ginsenoside Rg3+oxaliplatin significantly reduces PCNA and cyclin D1 expression, weakly binds cyclin D1 to CDK4, fails to inhibit cyclin D1-CDK4 complex formation, inhibits cell proliferation, and stimulates hepatoma cell SMMC-7721 apoptosis, thereby exerting anticancer effect [48].

### 3.2. 20(S)-Ginsenoside Rg3 and Doxorubicin

Some researchers study the ability of 20(*S*)-Rg3 to reduce autophagy as well as affect the chemosensitivity of HCC cell lines to doxorubicin and find that 20(*S*)-Rg3 may inhibit advanced autophagy by changing the expression of genes. Autophagy induced by adriamycin has a protective effect on HCC. Meanwhile, 20(*S*)-Rg3 treatment synergistically killed HCC cell lines with doxorubicin but is relatively nontoxic to normal hepatocytes. In addition, in addition to being well tolerated, 20(*S*)-Rg3 synergistically inhibits tumor growth in HCC xenografts in mice with doxorubicin. Rg3, as a new inhibitor of autophagy *in vivo*, has a good clinical application effect. Therefore, the combination of 20(*S*)-Rg3 and classical chemotherapy drugs may be a successful new technique to overcome chemotherapy resistance as well as improve chemotherapy efficacy [33].

### 3.3. Ginsenoside Rg3 and Sorafenib

Sorafenib has been approved by the Food and Drug Administration (FDA) as a first-line systemic therapy for advanced HCC; however, drug resistance to sorafenib usually affects its long-term efficacy [49]. 20(*S*)-ginsenoside Rg3 has been reported with significant anticancer effect to HCC and sensitizing HCC cells to chemotherapeutic agents [33, 50]. Sorafenib combined with 20(*S*)-ginsenoside Rg3 shows better results than the monotherapy group, the viability of HCC cells significantly decreased, and the apoptosis rate increased. The expression of PTEN, Bax, and cleaved caspase-3 increased, while the expression of phosphorylated PDK1 and phosphorylated Akt decreased. At the same time, the tumor volume and weight decreased in mice. These results suggest the synergistic anticancer activity of 20(*S*)-ginsenoside Rg3 and sorafenib by modulating PTEN/Akt signaling pathway in HCC [51]. Moreover, it has also been shown that combined treatment with Rg 3 and sorafenib significantly reduced cell viability, glucose consumption, lactate levels, and protein expression of HK2, PI3K, and Akt in HCC cells, alleviating hepatocellular carcinoma progression by regulating HK2-mediated glycolysis and PI3K/Akt signaling [52].

### 3.4. Ginsenosides and Transcatheter Arterial Chemoembolization

Transcatheter arterial chemoembolization (TACE) is the main therapy for the treatment of advanced liver cancer but is often limited for its complications [53]. By blocking tumor blood vessels, TACE induces a local hypoxic environment around hepatocellular carcinoma, activates vascular endothelial growth factor and epithelial growth factor, and subsequently induces angiogenesis and metastasis. However, TACE may cause serious adverse effects due to the toxicity of embolic materials and chemotherapeutic drugs [54]. Studies have shown that TACE combined with ginsenoside Rg3 can improve the disease control rate, objective response rate, and quality of life and alleviate nausea and vomiting, pain, hyperbilirubinemia, leukopenia, myelosuppression, thrombocytopenia, and *α*-fetoprotein in patients with HCC. Symptoms, combined with Rh2, can reduce the symptoms of thrombocytopenia, combined with CK, to relieve nausea and vomiting, fever, pain, and leukopenia, respectively. Combined use of ginsenosides can continuously improve the efficacy and safety of TACE treatment for liver cancer, and Rg3 is the first choice for combined use [55].

### 3.5. Ginsenoside Rg3 Nanoparticle Conjugation

To optimize the quick gastrointestinal passage and lower liver absorption rate of Rg3, the Fe@Fe3O4 nanoparticles are conjugated with ginsenoside Rg3 (NpRg3) to obtain nanomedicine with excellent coupling effect, which increases the water solubility and stability of ginsenoside and ginsenoside anticancer effect. It is found that the application of NpRg3 significantly increases the survival of dimethylnitrosamine-induced HCC model mice and eliminated HCC metastasis to the pulmonary. Importantly, NpRg3 administration alters gut microbiota structure and delays HCC-induced gut microbiota changes by at least 12 weeks during HCC progression. In addition to increasing *Bacteroidetes* and *Verrucomicrobia* abundance, NpRg3 also decreases *Firmicutes* abundance. Furthermore, NpRg3 may improve the pathophysiology of the ileocecal region and ultimately have a crucial function in HCC treatment. Additionally, the metabolomic profile progresses significantly throughout HCC progression, and NpRg3 administration corrects the tumor-dominant metabolomics. Taking NpRg3 reduces 3-indolepropionate and urea but increases free fatty acids. In conclusion, NpRg3 application contributes to the remodeling of the related imbalance network between gut microbiota and liver tissue metabolism during liver cancer treatment, which provides a new idea for tumor treatment [30, 56].

### 3.6. Ginsenoside Rg3, *Ganoderma lucidum* Polysaccharide, and Oridonin Self-Microemulsifying Drug Delivery System (RGO-SMEDDS)

In addition to the combination with chemotherapy drugs, ginsenoside can be combined with *Ganoderma lucidum* polysaccharide (GLP) and oridosin as therapeutic agents. These three plant monomers have a significant effect in antiangiogenesis, immunological activation, and apoptosis induction, respectively. Nevertheless, limited solubility and poor absorption impede their practical utilization significantly. To solve these issues, we developed a unique medication self-microemulsification delivery method for Rg3, GLP, and oridonin (RGO-SMEDDS). It was discovered that treatment restored immunological function by inhibiting the production of immunosuppressive cytokines and M2-polarized macrophages and decreased angiogenesis by downregulating vascular endothelial growth factor and its receptor. Inhibition of epidermal growth factor receptor EGFR/AKT/epidermal growth factor receptor protein kinase B/glycogen synthase kinase 3 (GSK3) signaling pathway retarded proliferation. The findings imply that RGO-SMEDDS is a potential therapy for HCC [57].

## 4. Conclusion

**Figure 2:**
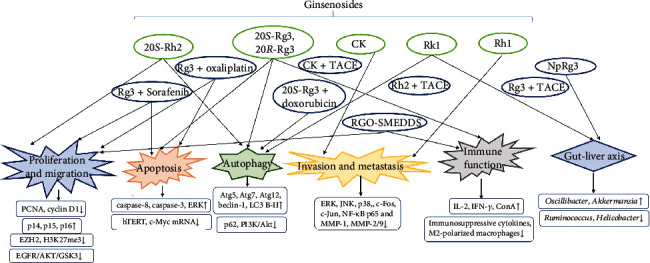
The various mechanisms behind the anticancer property of ginsenosides.

In summary, there have been many reports on various mechanisms of ginsenosides in HCC therapy (Figure 2), including proliferation and migration, apoptosis, autophagy, regulated invasion and metastasis, immune function, gut-liver axis, and combination therapy, which provide new targets and ideas for the treatment of HCC. In particular, the use of ginsenosides combined with chemotherapy for HCC can improve the sensitivity of chemotherapy drugs and reduce the side effects of chemotherapy, which has attracted people's attention to its combination with conventional chemotherapy. Content of ginseng saponins in *Panax ginseng* varieties, however, is rare, and most of the ginseng saponin monomers have low solubility in water; elimination rate is fast, low bioavailability inherent disadvantages are clinical; therefore, we still need to conduct further clinical trials of ginsenosides and extensive pharmacokinetic study, with the use of natural active ingredients to develop safer, more efficient, and low-toxicity drugs.

## Figures and Tables

**Table 1:** Study on the therapeutic effects and mechanisms of ginsenosides on HCC.

Cell lines/mice	Types of ginsenosides	Physiological effects	Related mechanisms	Refs.
SMMC-7721and SK-Hep-1 cells	Rg3	Inhibits the proliferation and invasion	Regulates long noncoding RNA HOX antisense intergenic	[20]
HepG2 cells	20(*S*)-Rh2	Reduces the growth and migration ability	STAT3/VEGF pathway	[21]
HepG2 and SK-Hep-1 cells	20(*S*)-Rh2	Arrests at the G0/G1 phase and inhibited cell growth	Disturbs the HSP90A-Cdc37 chaperone system	[22]
HepG2 and Hep3B cells	20(*S*)-Rh2	Suppresses proliferation and migration	Regulates CDKN2A-2B gene cluster transcription	[23]
HepG2 cells	Rk1	Inhibits telomerase activity and induces apoptosis	Decreases hTERT and c-Myc mRNA	[29]
Hep1-6 cells/C57BL/6 mice	Rg3	Induces apoptosis	Inhibits the activation of microtumor vessel formation *in vivo*	[30]
SK-Hep1, HepG2, A549, and H322 cells	20(*S*)-Rg3	Inhibits autophagy	Changes in gene expression and activates of the CHOP transcription factor	[33]
HepG2 and Huh7 cells/NOD/SCID mice	Rh2	Regulates autophagy	*β*-Catenin signaling	[34]
HepG2 cells	Rh1	Suppresses matrix metalloproteinase-1 expression	Inhibits of activator protein-1 and mitogen-activated protein kinase signaling pathway	[37]
MHCC97-H	CK	Inhibits of spontaneous metastatic growth of HCC cells	Translocation of nuclear factor-*κ*B p65 and the reduction of MMP-2/9	[38]
H22 cells/KM mice	Rg3	Improves the host's cellular immunity	N/A	[40]
C57BL/6J mice	Rk3	Targets the gut-liver axis	N/A	[45]

**Table 2:** Combined applications of ginsenosides against hepatocellular carcinoma.

Types	Cell lines/mice/patients	Ginsenosides and compounds	Physiological effects	Related mechanisms	Refs.
	SMMC-7721 cells	Rg3 and oxaliplatin	Inhibits proliferation and promotes apoptosis	Downregulates PCNA and cyclin D1-CDK4 compound	[48]
	SK-Hep1, HepG2, A549, and H322 cells	20(*S*)-Rg3 and doxorubicin	Inhibits autophagy	Changes in gene expression and activates of the CHOP transcription factor	[33]
*In vitro*	HepG2 and Huh7 cells	20(*S*)-ginsenoside Rg3 and Sorafenib	Suppresses proliferation and induces apoptosis	Modulates PTEN/Akt signaling pathway	[51]
	HepG2 and Bel7404 cells	Ginsenoside Rg3 and sorafenib	Reduces cell viability, glucose consumption, and lactate levels	Regulates the HK2-mediated glycolysis and PI3K/Akt signaling pathway	[52]
	HepG2 and Huh7 cells	20(*S*)-ginsenoside Rg3 and sorafenib	Suppresses HCC cell proliferation and induces apoptosis	Modulates PTEN/Akt signaling pathway	[51]

*In vivo*	C57BL/6 mice	Nanoparticle conjugation of ginsenoside Rg3	Remodels of unbalanced gut microbiota and metabolism	N/A	[56]
	C57BL/6 mice	Rg3, *Ganoderma lucidum* polysaccharide, and oridonin	Restores immune function, reduces angiogenesis, and retards proliferation	Inhibits the epidermal growth factor receptor EGFR/AKT/epidermal growth factor receptor/protein kinase B/GSK3 signaling pathway.	[57]

In clinical	371 HCC patients	Ginsenoside Rg3 and sorafenib	Reduces cell viability, glucose consumption, and lactate levels	Regulates the HK2-mediated glycolysis and PI3K/Akt signaling pathway	[52]
	18 RCTs with 1308 HCC patients	Ginsenosides and TACE	Continuously benefits the efficacy and safety of TACE in HCC treatment	N/A	[55]
